# Deep-Sequencing of the Peach Latent Mosaic Viroid Reveals New Aspects of Population Heterogeneity

**DOI:** 10.1371/journal.pone.0087297

**Published:** 2014-01-30

**Authors:** Jean-Pierre Sehi Glouzon, François Bolduc, Shengrui Wang, Rafael J. Najmanovich, Jean-Pierre Perreault

**Affiliations:** 1 Département d’informatique, Faculté des Sciences, Université de Sherbrooke, Sherbrooke, Québec, Canada; 2 Département de biochimie, Faculté de médecine et des sciences de la santé, Pavillon de Recherche Appliquée au Cancer, Université de Sherbrooke, Sherbrooke, Québec, Canada; Centers for Disease Control and Prevention, United States of America

## Abstract

Viroids are small circular single-stranded infectious RNAs characterized by a relatively high mutation level. Knowledge of their sequence heterogeneity remains largely elusive and previous studies, using Sanger sequencing, were based on a limited number of sequences. In an attempt to address sequence heterogeneity from a population dynamics perspective, a GF305-indicator peach tree was infected with a single variant of the *Avsunviroidae* family member *Peach latent mosaic viroid* (PLMVd). Six months post-inoculation, full-length circular conformers of PLMVd were isolated and deep-sequenced. We devised an original approach to the bioinformatics refinement of our sequence libraries involving important phenotypic data, based on the systematic analysis of hammerhead self-cleavage activity. Two distinct libraries yielded a total of 3,939 different PLMVd variants. Sequence variants exhibiting up to ∼17% of mutations relative to the inoculated viroid were retrieved, clearly illustrating the high level of divergence dynamics within a unique population. While we initially assumed that most positions of the viroid sequence would mutate, we were surprised to discover that ∼50% of positions remained perfectly conserved, including several small stretches as well as a small motif reminiscent of a GNRA tetraloop which are the result of various selective pressures. Using a hierarchical clustering algorithm, the different variants harvested were subdivided into 7 clusters. We found that most sequences contained an average of 4.6 to 6.4 mutations compared to the variant used to initially inoculate the plant. Interestingly, it was possible to reconstitute and compare the sequence evolution of each of these clusters. In doing so, we identified several key mutations. This study provides a reliable pipeline for the treatment of viroid deep-sequencing. It also sheds new light on the extent of sequence variation that a viroid population can sustain, and which may give rise to a quasispecies.

## Introduction

Viroids are plant-restricted infectious agents composed of a 245–401 nucleotide circular RNA genome (for a review see [1]). They are non-encapsidated and do not code for any protein. Their genomes possess sufficient information to take over the plant’s transcriptional machinery and produce progeny that spread throughout the entire plant resulting in specific diseases [2]. They are divided into two families based on the presence or absence of a conserved central region (CCR) in their genome. The *Pospiviroidae* family is characterized by the presence of a CCR, and its members accumulate in the nucleus. Conversely, the *Avsunviroidae* family is characterized by the absence of CCR. Additionally, its members self-cleave via a *cis*-acting hammerhead motif during their replication cycle which takes place in the chloroplast.

It is still not known how viroids elicit pathogenesis. However, recent studies have focused on the implication of the RNA interference machinery. For example, it has been demonstrated that a hairpin derived from *Potato spindle tuber viroid* (PSTVd) alone can induce the symptoms associated with viroid infection when it is introduced into tomato plants [3]. Moreover, *Peach latent mosaic viroid* (PLMVd) variants inducing the peach calico disease, as well as the Y-satellite RNA of *Cucumber mosaic virus* (CMV), can induce symptoms following the interaction of viroid-siRNA with a specific host mRNA, thus silencing the targeted genes through an RNA-induced silencing complex (RISC) mediated degradation [2], [4]. In the case of PLMVd, the primary, rather than the secondary, structure mediates the symptoms observed during the peach calico disease through the binding of viroid small interfering RNAs with specific host mRNAs, resulting in the downregulation of the targeted RNA. Furthermore, data obtained with PSTVd have shown that the closing of one of its specific loops (i.e. loop 6), after a substitution of only 3 nucleotides, abolishes the systemic trafficking of this viroid in *Nicotiana benthiamana* [5], [6], suggesting a correlation between its sequence and/or its structure and the infection. Thus, accurate knowledge of a viroid’s sequence, and of the sequence heterogeneity of mutant sequences generated following infection, is a key aspect in the elucidation of its pathogenesis.

Since their discovery in 1971 [7], a relatively large number of viroid sequences have been reported in public databases such as the Subviral one [8]. Currently, there are 2,800 sequences, representing a total of 34 different species, demonstrating the degree of intra-host genetic variation that exists in viroid populations. *Avsunviroidae* viroids like PLMVd are replicated by a proofreading-deficient DNA-dependent RNA polymerase that is redirected to use RNA as template [9], [10]. As a consequence, viroid mutation rates are the highest (2.5×10^−3^ per site per replication cycle) reported to date for a biological entity [9]. More than 300 distinct sequences of PLMVd have been reported to date. These two facts led several investigators to claim that PLMVd, and more generally viroids, can be represented as clouds of related RNA sequences in the multi-dimensional space of sequences that can be designated as quasispecies [11]. In such a space, each point represents one particular sequence.

All PLMVd sequence variants reported to date have been cloned from total RNA isolated from a single tree (or a group of trees) using small-scale sequencing. The genetic variability of the sequences that may be found in a single host has been addressed by Ambros and coworkers in two reports [12], [13] using Sanger sequencing through the respective analysis of 29 and 36 clones of PLMVd. These reports show that PLMVd sequences can be clustered into families. This assumption, however, was based on the analysis of a limited number of sequences i.e. 29 and 36 different clones, respectively. With the advent of high-throughput sequencing technologies (HTS) it is now possible to reconsider the question of viroid sequence heterogeneity, based on a relatively large-scale number of sequences. This should provide additional support to the conclusions of previous studies. Importantly, the relatively small size of a viroid’s genome enables a single HTS run, using the 454 technology, to determine full-length sequences [14]. Clearly, HTS is a powerful tool with which to investigate the sequence heterogeneity of a population of viroid molecules [15]. In this study, we report a pipeline that permits to take a “snap-shot” of a viroid population 6 months post-infection. We designed an experimental strategy combining HTS and a clustering algorithm, based on a top-down divisive approach without recourse to multiple alignments. We harvested a total of 3,939 novel sequences, including a functional natural-hammerhead sequence. To our knowledge, this is the most comprehensive report to date, by over an order of magnitude in terms of novel sequences. The systematic analysis of this large collection of sequences revealed several new features pertaining to the evolution of PLMVd genetic heterogeneity and sequence compositions themselves.

## Results

### Deep-sequencing of Circular PLMVd Conformers

**Figure 1:**
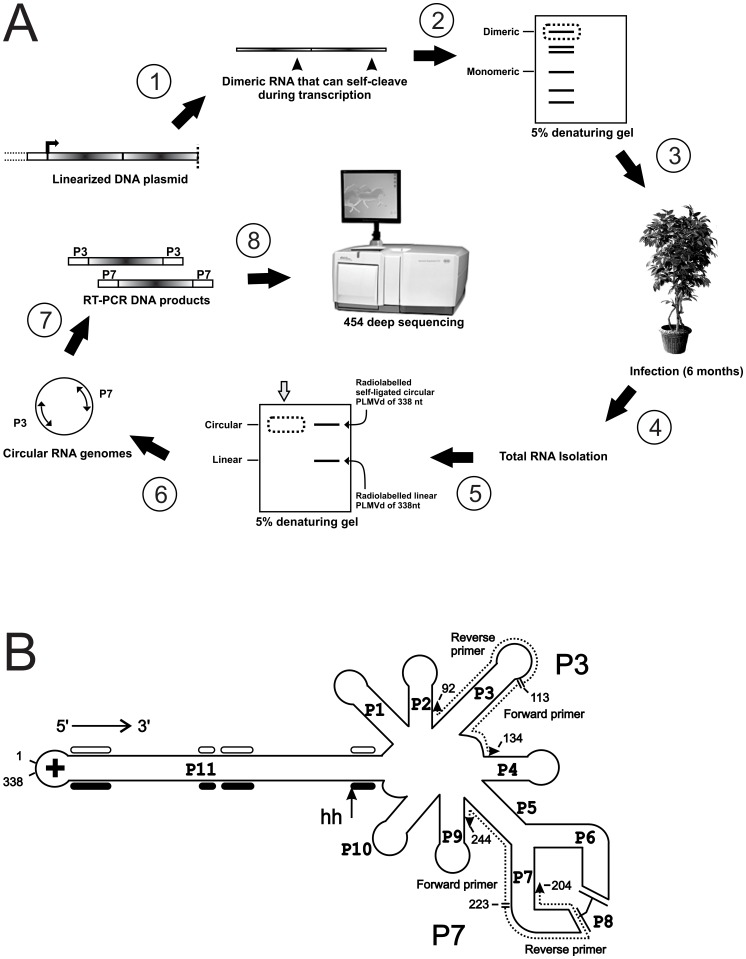
Experimental scheme. (A) A plasmid containing a dimeric head-to-tail arrangement of the sequence of PLMVd.282 was digested with *SpeI* and then used in an *in vitro* transcription reaction (step 1). The resulting dimeric transcript was isolated by excision following migration on a denaturing gel (step 2) (see dotted line on the gel). This dimeric viroid RNA was slash-inoculated into one GF-305 peach tree (step 3). Six months post-infection, total RNA was isolated from leaves (step 4) and fractionated on a 5% denaturing gel (step 5). Radiolabelled 338 nt self-ligated monomeric circular and linear monomer PLMVd RNAs were also electrophoresed as markers on the gel. The circular conformers were isolated from the infected cell RNA by excision of the gel band (dotted line) corresponding to the radiolabelled circular PLMVd from the gel (step 6). RT-PCR amplification was then performed using two primer pairs (P7 and P3) (step 7) prior to deep-sequencing of the PCR products (step 8). (B) Both forward and reverse P3 primers bind to the region from nucleotides 92 to 134, while the P7 primers bind to the region from nucleotides 204 to 244. The four primers are represented by dotted lines here. Each stem (P1 to P11) is indicated, as is the hammerhead self-cleavage site of the (+) polarity strand (arrow). The black and open boxes below and above the P11 stem identify the regions that compose the hammerhead core of the (+) and (−) polarity strands, respectively.

In an attempt to assess the extent of genetic heterogeneity found within a viroid population, we performed a simple infection experiment consisting of the slash inoculation of a single PLMVd variant into a single peach tree. Six months post-infection, the viroid’s genomic circular conformer was isolated from the total RNA and then amplified before being completely sequenced. More specifically, a dimeric head-to-tail transcript of the PLMVd.282 variant (accession number: DQ680690), which is referred to as the “parental sequence” in this report, was used to infect a GF-305 indicator cultivar peach tree. This 337-long variant, which we identified in a previous sequencing study [16], bears one additional adenosine between nucleotides 107 and 108. It was shown to be infectious when inoculated into the GF-305 cultivar over the course of inoculation experiments performed with several other PLMVd variants (data not shown). Thus, we used this particular variant because it was known to be infectious and already available to us. During transcription of the DNA template containing the head-to-tail insert of the viroid, the hammerhead self-cleaving motifs do not cleave to completion. The uncleaved dimeric-RNA product was used for the slash-inoculation. Six months post-infection, the circular conformers were purified from the total RNA of infected peach tree leaves using a denaturing polyacrylamide gel (Figure 1A). Northern blot hybridization of RNA samples previously separated by native gel (i.e. without 8 M urea) electrophoresis confirmed that only circular conformers were recovered (data not shown). Subsequently, we performed RT-PCR amplification of the recovered circular RNA species, followed by deep-sequencing (Figure 1A). Because RT-PCR amplification generates no information from the regions bound by the primers, we used two pairs of primers complementary to both the P7 (positions 204 to 244) and P3 stem (positions 92 to 134) regions, which are believed to be highly conserved (Figure 1B). Together, both sets of primers – specific to the (+) polarity strand – provided information for the entire viroid genome.

The sequencing step generated two libraries representing a total of 787,678 reads which were filtered as described in the Materials and Methods Section (see also Table 1 for both the P7 and the P3 libraries). Filtering allowed the removal of all reads missing a primer region or containing ambiguous positions designated as “N” and reads shorter than 323 nts. An additional final step removed 116 unique reads with less than 70% homology to the PLMVd.282 parental sequence, for a total of 227 removed reads. All were rRNA, amplified by the P7 primers. From these filtering steps, 49.4% and 38.5% of the reads of the P7 and P3 libraries, respectively, were kept for further analyses. This provided a total of 339,148 reads corresponding to 56,804 unique sequences (Table 1) which were all longer than 323 nt considering the primer regions. Specifically, over 99% and 97% of sequences from the P3 and P7 libraries, respectively, were 337 nt +/−2 nt in length, whereas the parental sequence was 338-nt long. The other sequences were only slightly smaller or longer but always within the range previously observed for PLMVd i.e. 335 to 350 nucleotides in length. Indeed, this is a good indication of the quality of the sequencing.

**Table 1:** Analysis of the reads for both the P7 and P3 libraries obtained following deep-sequencing of the circular conformers RT-PCR amplified from the total RNA of the infected tree.

Library	# of unfiltered reads	After filtering		After error correctionby KEC		After thresold removal	
		Total # ofreads	# of unique reads	Total # ofreads	# of uniquereads	Total # ofreads	# of uniquereads
**P7**	329,094	162,584	30,890	158,610	17,622	136,906	2,087
**P3**	458,584	176,564	25,914	172,829	14,358	155,053	1,852
**Total**	**787,678**	**339,148**	**56,804**	**331,439**	**31,980**	**291,959**	**3,939**

### Refinement of the Sequence Libraries Based on Error-correction and Phenotypic Character

The libraries resulting from the filtering step are comprised of completed and defined reads. That said they may also include reads containing mutations introduced, either during RT-PCR reactions or deep-sequencing, which are also called “technical” mutations. Such mutations therefore do not reflect natural variations and should be removed prior to any significant analysis. To achieve our goal we decided to use an error-correction algorithm combined with a phenotypic character based on hammerhead self-cleavage.

Firstly, in order to remove “bad” reads or correct errors within reads, we used an error correction algorithm optimized for viral amplicons and obtained through 454-deep sequencing called KEC (K-mer-based Error Correction) [17]. The filtered reads from both libraries were analyzed by KEC and the summary is shown in Table 1. After error-correction, the number of reads decreased by 2.1% and 2.4% for P3 and P7 libraries respectively. Interestingly, KEC algorithm reintroduced corrected reads shorter than 323 nts although small reads had previously been removed during the filtering step (see above). Thus newly introduced short reads were removed, prior to further analysis, because they do not represent viable amplicons.

**Figure 2:**
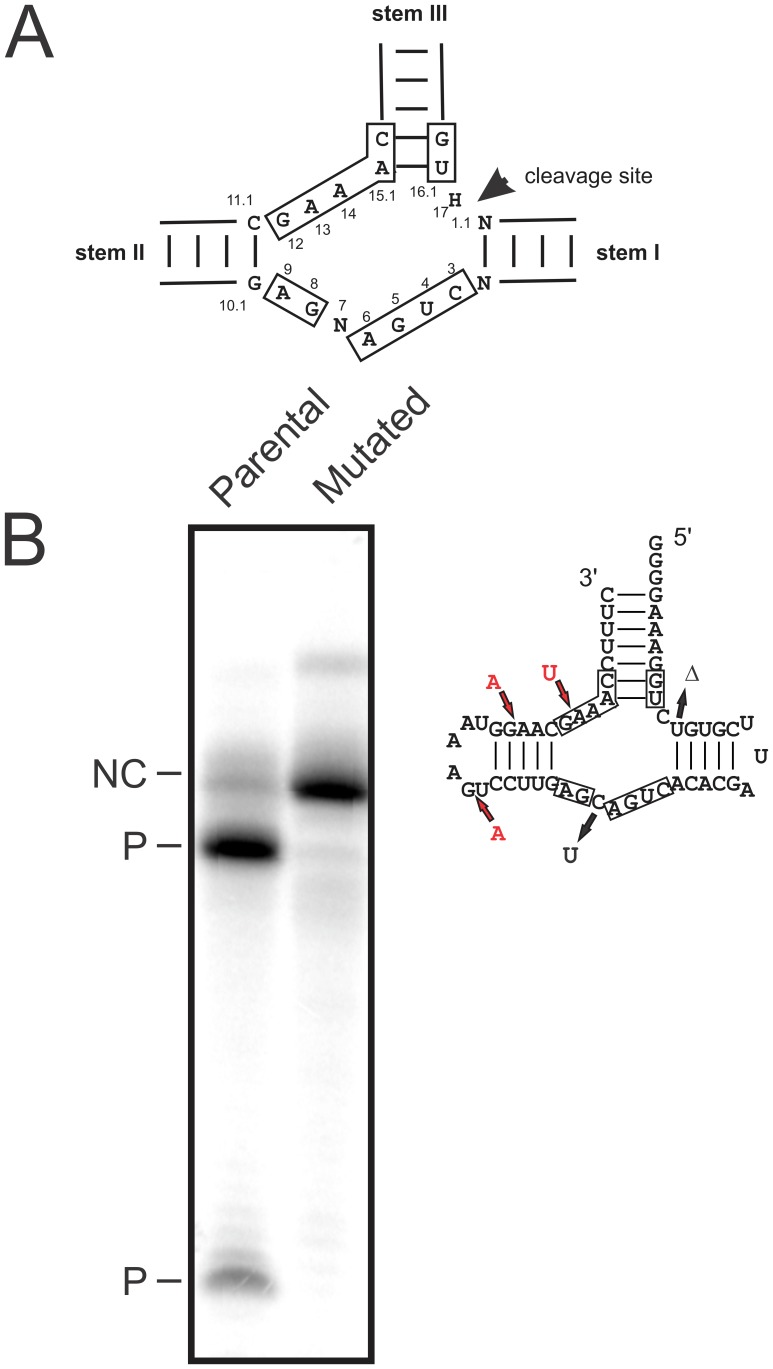
*In vitro* cleavage assay of minimal versions of hammerhead sequences. (A) Schematic representation of the consensus sequence of the catalytic core of the hammerhead sequences. The core nucleotides, identified by the boxes, are flanked by the helical stems I-III. The numbering is according to Hertel et al. [33]. (B) Left side: the cleavage assay for the parental and the mutated hammerhead found in reads of 4 occurrences was monitored during 60 min transcription reactions at 22^o^C, and was analyzed on a 15% denaturing gel. The non-cleaved full-length transcripts are indicated by the letters NC, while the cleaved products are identified by the letter P. Right side: schematic representation of the tested hammerhead motif found in a read of 4 occurrences in the P7 library. The mutations relative to the parental sequence are identified by arrows where the red arrows denoted insertions.

Secondly, since technical mutations can still occur in the libraries after KEC software analysis, we applied a second level of correction by investigating the hammerhead-core composition found within each read. Previous reports have shown that changes to the catalytic core composition of the hammerhead self-cleaving motif (see the 13 boxed nucleotides for a strand of one polarity depicted in Figure 2A) significantly affect cleavage ability [18]–[20]. The very highly-conserved nucleotides of the catalytic core were used to eliminate reads with variations that still occur. In this study, we specifically isolated circular conformers to build both libraries. Thus, the variants identified from our data emerged from molecules able to self-cleave. Rolling-circle replication generates longer-than-unit conformers that self-cleave into monomers to be ligated thereafter. Thus, we reasoned that sequences in which the hammerhead-core nucleotides are mutated in a way that impairs self-cleavage must logically arise from technical mutations. Therefore, we used the self-cleavage phenotype to identify such technical mutations and combed through our libraries in search of sequences bearing mutations in the conserved hammerhead-core nucleotides.

Analysis of the reads from the P3 library failed to highlight any mutations in the hammerhead-core nucleotides, while one mutation was detected in the P7 library. The same mutation, found in reads of 1, 2 and 4 occurrences, is a uridine insertion between G12 and A13 (see nucleotide numbering in Figure 2A). Here the number of occurrences is defined as the number of times that a specific read (or nucleotide sequence) was found in a library. The mutated minimal hammerhead sequence was tested in a self-cleaving reaction during *in vitro* run-off transcription of the cognate DNA template (Figure 2B). The cleavage efficiency of the resulting radiolabelled *cis*-acting RNA transcript was compared to the parental minimal hammerhead sequence. The hammerhead motif of the parental sequence was considered as the reference sequence, exhibiting >90% self-cleavage following one-hour incubation, while the mutated hammerhead failed to generate any detectable cleavage activity (Figure 2B). As mentioned above, we reasoned that reads containing mutations that impair self-cleavage should be removed as they logically arise from technical mutations rather than natural selection. Thus, all reads with 4 occurrences or less were discarded and did not undergo any further analysis since they were considered to contain technical mutations, not only in the hammerhead region, but anywhere else around the molecule. The removal was also performed for the P3 library although no mutation was detected in the hammerhead-core nucleotides. Removal of the reads with less than 4 occurrences resulted in a reduction of the total number of reads by 11.9% to 291,959 reads. This in turn reduced the number of unique sequences by 87.7% to 3,939 reads. It is important to note that approximately 65–67% of removed sequences were unique occurrences, i.e. single-occurrence reads (see Figure S1). This threshold may appear overly stringent, but we deemed more appropriate to remove a larger number of reads than to work with experimental artefacts. That said, mutations located outside of the hammerhead-core nucleotides may also impair self-cleavage. When sequences from both the P7 and P3 libraries were compared, those composing the P7 library displayed more heterogeneity. Indeed, P7 nucleotide diversity was 1.60 and greater than P3 diversity which was 1.39. Nucleotide diversity represents the proportion of nucleotide differences per sites between sequences relative to sequence frequencies [21]. It is interesting to note that the average number of mutations, relative to the parental sequence (PLMVd.282) used for inoculation, was 6.4, and that it ranged from 3 to 22 mutations in the P7 library. Although the average number of mutations was lower in the P3 library (4.6 mutations), the range (2 to 51 mutations) was broader (see Figure S2). Indeed, in the P3 library, a relatively small number of sequences (121 sequences) contained between 36 and 51 mutations. When these sequences are excluded, all of the remainder displayed between 2 and 19 mutations. The higher average number of mutations observed in the P7 library most likely originates from the P3 region. Sequencing data from this region are missing in the P3 library because the primers used also anneal to this region during the cloning step. Alternatively, it may also suggest that the region bound by the P7 primers (P7 stem) is less heterogeneous.

### Analysis of 3,939 PLMVd Sequence Variants

**Figure 3:**
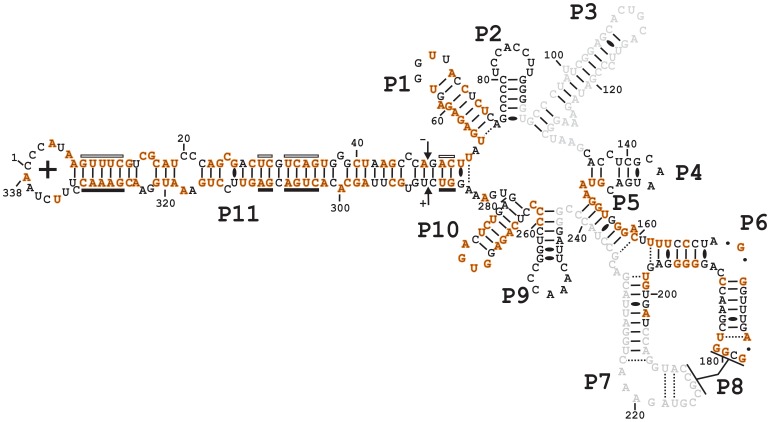
Nucleotide conservation. Accepted secondary structure of PLMVd (+) showing, in orange, the nucleotides that are 100% conserved when the data from both libraries are merged. The shaded nucleotides represent the regions bound by the primers.

Taken together, both libraries included a total of 3,939 novel PLMVd variants that were deposited in both the NCBI (accession numbers KF866387 to KF870325) and Subviral databases. The initial tree-inoculate parental sequence was not found in either of the libraries despite the use of high-throughput sequencing to detect the highest number possible of different sequences. This confirms a similar observation reported previously, based however on a limited number of sequences obtained by Sanger sequencing [12], [13]. The majority of key mutations found in high-occurrence reads were the result of substitutions, with a lesser number due to deletions and insertions. Mutations were found to be located all around the PLMVd genome. Merger of the sequences from both libraries revealed that 118 out of 254 positions remained perfectly conserved (100% conserved). These numbers do not take into account the positions of the two regions covered by the primers. Thus, we found a 46.5% level of conservation (see Figure 3). The most highly conserved region of PLMVd is the left hand region comprising P1, P10 and P11 stems displaying conservation levels of 70.6%, 50.0% and 67.4% respectively. Included in the conserved regions is the P10 loop that can be defined as a GNRA tetraloop (GUGA in this case) which is the only conserved loop of the viroid. Interestingly, one strand of the P5 stem is also well conserved (nucleotides 149–160), although located in the right-hand region of PLMVd. Unfortunately, no sequencing data was recovered for the other P5 strand since the primers of P7 library bind precisely this region. However, data based solely on the P3 library suggest that nucleotides along both strands of the P5 stem are well conserved.

When comparing at the sequence variation in the separate libraries, data from P3 and P7 showed that 178 nucleotides out of 295 and 212 positions out of 297 respectively (excluding the region covered by the primers) remained perfectly conserved, yielding respective levels of conservation of 60.3% and 71.4%. Finally, the two most abundant reads found occur 14,848 and 20,483 times in P7 and P3 respectively (see Figure S1). Together, these data show that PLMVd is clearly a mutation-prone RNA genome.

### Clustering of PLMVd Sequences using a Hierarchical Algorithm

In order to analyze the important number of reads, and to track the evolution of PLMVd heterogeneity following inoculation, we used a hierarchical (DHCS) algorithm [22] designed for the general purpose of clustering categorical sequences. A unique advantage of DHCS is that it relies on an advanced statistical model called “Conditional Probability Distribution” (CPD) to characterize a group of sequences, and to estimate similarity between an individual sequence and those of the group. This allows DHCS to bypass sequence alignment for similarity computation purposes. DHCS is therefore able to deal with various kinds of biological sequences, regardless of whether or not they are simple to align. As the first top-down divisive hierarchical algorithm for sequence clustering, DHCS improves several aspects of the clustering process. It bypasses comparisons of individual sequences in the estimation of pair-wise similarities during the clustering process, and yields statistically significant clusters. It also offers an automatic method for estimating the number of clusters, and permits the generation of a hierarchy of clusters. In [22], it was shown that DHCS is effective for the clustering of various sequence types including protein sequences.

Technically, DHCS owes its clustering capacity to a two-tier Markov model. The above-mentioned CPD model [23]–[25] used in DHCS to represent a group (cluster) of sequences is in fact a general Markov model of variable orders. It has the potential to explain the sequence generation process of a group by exploring significant motifs common to the group and to naturally integrate these motifs in similarity estimation. That said, the application of the CPD model to clustering presents computational difficulties in initializing clusters and in estimating the parameter values of the CPDs. The two-tier Markov model developed in DHCS overcomes these difficulties by using a first-order Markov model which yields a vector representation of each sequence in a group. The vector representation facilitates the splitting of the group as a solution to an optimization problem. The groups obtained from the optimized splitting are then used to initialize the CPDs, and are then optimized via a final refinement phase.

**Figure 4:**
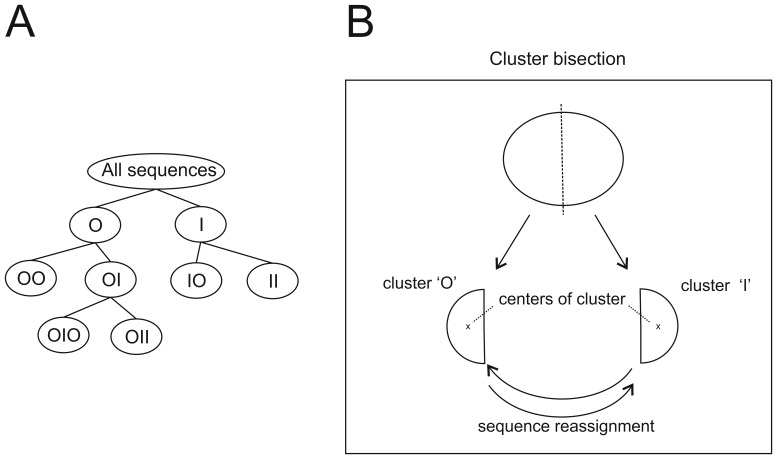
Generation of clusters by the DHCS algorithm. (A) All of the sequences in each library were clustered using the DHCS algorithm developed by Xiong et al, [22]. (B) During the clustering, the sequences are grouped according to their relative similarities and each sequence is tested with the center of each cluster (I or O). It is still possible at this point that a specific sequence may be retrieved in another cluster. This step is repeated until the clusters become stable and the distance between each sequence and the center is minimal.

The two-tier Markov model is recursively used in DHCS to create the hierarchy of clusters. Starting with the entire set of sequences, DHCS successively bisects each cluster, thereby generating a binary tree. The nomenclature used is as follows: the name of each child node or cluster obtained after splitting is formed by adding ‘O’ for the left cluster and ‘I’ for the right cluster. For instance, “OOO” represents the left cluster from the bisection of cluster “OO” (Figure 4A). Each bisection (Figure 4B) is initialized by using Fuzzy-Multiple Component Analysis (F-MCA) [26], [27] on the vector representation of the sequences produced by the first-order Markov model. The two groups are then optimized according to this first-order Markov representation, and the Chi-square distance between each sequence and each group is then used, producing two refined clusters. Finally, a variable-order CPD model for each of the two clusters is built depending on the motifs discovered from each cluster and on their statistical significance.

**Figure 5:**
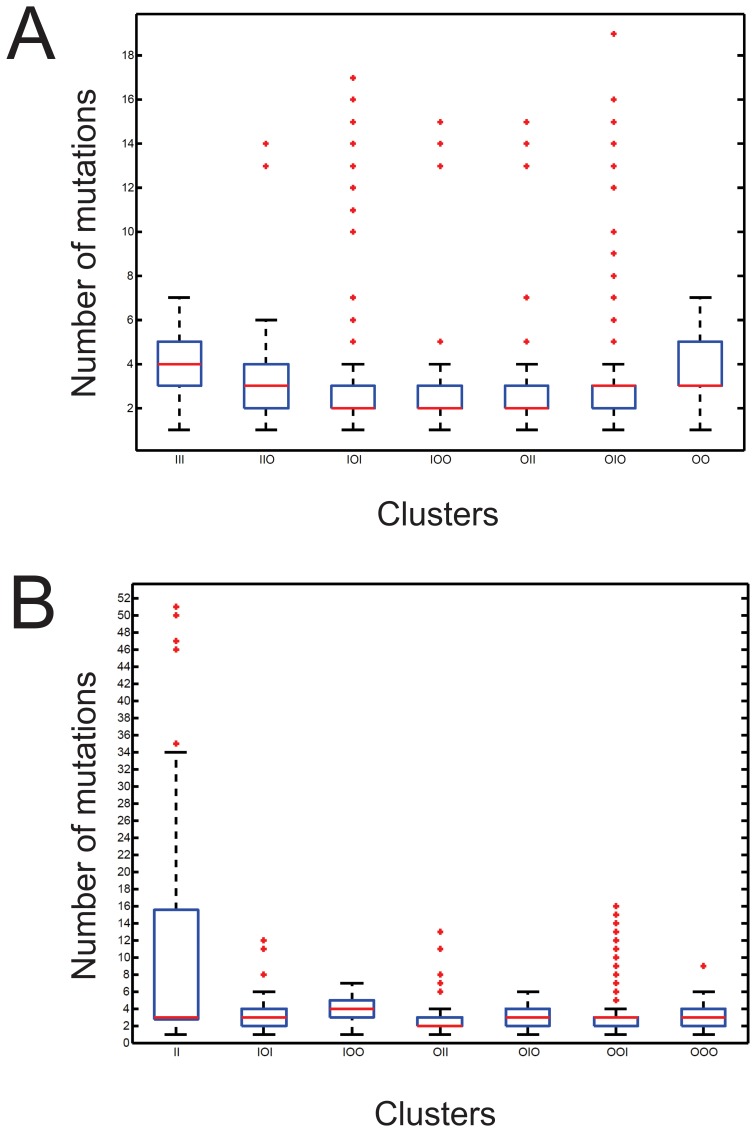
Dispersion of the variants relative to the representative sequence of each cluster. Box-and-whisker diagrams showing the dispersion of the variants relative to the representative sequence in each cluster of the P7 (A) and P3 (B) libraries. For each cluster, the blue box contains 50% of all of the sequences in the cluster. The red line shows the median of the number of mutations relative to the representative sequence of the cluster, and the little red crosses represent outliers of the cluster.

Application of the DHCS algorithm led to the grouping of the reads into 7 clusters for each of the P7 and P3 libraries (Figure 5). In the next two subsections, we provide biological interpretations of these results based on mutations. Here, we report on the evaluation of clustering results, according to within-cluster cohesion and between-cluster separation. Since DHCS is a similarity-based clustering algorithm, the cluster cohesion/separation analysis will be based on average within-cluster and between-cluster similarities.

First, given a sequence 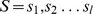 and a cluster Δ, the following similarity measure is used [25]:
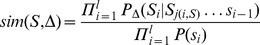
where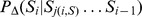 is the probability of observing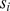 given its preceding subsequence of 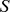 which is calculated by the CPD model of the cluster Δ, and 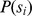 is the probability of the occurrence of symbol 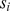 out of the whole set of sequences. 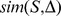 describes in fact the likelihood that 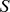 is generated by the statistical model of the cluster Δ. From this similarity measure, the within-cluster cohesion and between-cluster separation are defined as follows



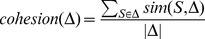


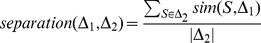



In other words, 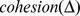 is the average similarity between each sequence of the cluster and the model of the cluster, while 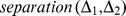 is the average similarity between each sequence of one cluster 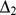 and the model of another cluster 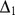. Note that the above *separation* is a non-symmetric measure, but can be easily transformed to a symmetric one by 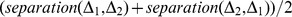. The two following tables show the results of cohesion/separation analysis of the clusters generated for the datasets P3 and P7 (see Figure S3). In these two tables, the diagonal elements are cohesion values and off-diagonal elements are separation values where each row cluster corresponds to the reference cluster 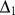
in the separation measure. As can be noticed, the cohesion is at least one order larger than the average separation for each cluster. This analysis therefore confirms the quality of the clustering obtained by DHCS in terms of within-cluster cohesion and between-cluster separation.

### Results Analysis According to Mutation Frequencies

Analysis by sequence comparison was then performed and “representative sequence” of each cluster was identified. The representative sequence is at the center of the swarm i.e. the sequence possessing the minimum total dissimilarity to all sequences of the same cluster. All sequences of a given cluster were first multiple aligned according to order of length and the resulting shorter sequences displaying “-” at certain positions were then aligned pairwise. For each sequence, mutational distances to other sequences of that cluster were calculated in terms of the median number of mutations. Finally, the sequence with the minimum number of mutations with respect to all other sequences was retained as the representative sequence of the cluster. This analysis revealed that all clusters possess a median number of mutations relative to the representative sequence between 2 and 4 (Figure 5).

Next, we attempted to identify those mutations that distinguished clusters from one another, and to recapitulate the sequence of events leading up to the observed differences in cluster “fitness” (explained below). To do so we performed a multiple alignment of the representative sequences of each cluster of both libraries against the parental sequence originally introduced into the plant (see Figures S4 and S5). In this study, the biological fitness of a given sequence is approximated by the frequency with which it appears in the population, as seen through the lens of the sequenced data. Thus, high-occurrence reads are considered fitter than low-occurrence reads. This is not the first time that the notion of “fitness” is applied to a viroid. It has been used previously in a study based on a limited number of variants determined by Sanger sequencing [11]. In this study, encompassing a total of 3,939 different reads, the “fitness” of a cluster takes on its full meaning. Sequence proximity i.e. the number of mutation differences relative to the parental sequence, was used as a molecular clock. It is reasonable to imagine that different positions in the viroid sequence evolved with different mutational rates, due to functional and structural constraints. However, in this analysis, the relative sequence of evolutionary events within the study population was ordered based on the distance between variants as measured in sequence space.

**Figure 6:**
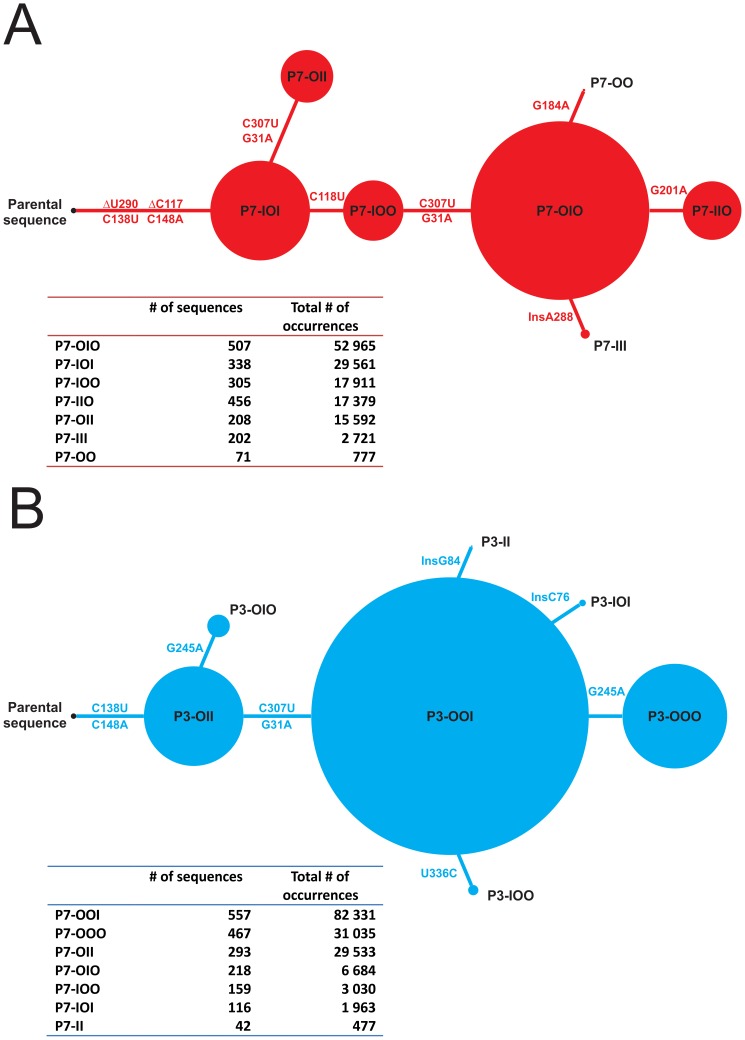
Clusters and discriminating mutations. (A) Scale schematic representation of the clusters (shown here as a tree) found in the P7 library, starting with the parental sequence. Firstly, the representative sequences of each cluster were multiple aligned with the parental sequence to identified mutations that discriminate the clusters. Secondly, the representative sequences are sorted according to the number of mutations they bear regarding the parental sequence. Each cluster is represented by a red circle for which the size is determined by the total number of occurrences contained in the cluster. Also, clusters are linked with lines showing the key mutations found within the representative sequences of each cluster. Below the scheme, a table compiling the details of each cluster regarding the number of sequences, the total number of occurrences and the fraction (%) that account for the representative sequence of each cluster. (B) Same as in A, but for the P3 library using blue circles.

We created a scaled drawing that connects cluster size to chronological appearance of mutations post-inoculation with the parental sequence. We used the representative sequence of each cluster to highlight the key mutations that distinguish it from each of the other clusters. Thus, we established the concept of a mutational “distance” connecting all of the different clusters up to the parental sequence. The smaller the number of mutations born by the representative sequence of a given cluster with respect to the parental sequence, the “older” and therefore the closer the cluster should be to the parental sequence. Conversely, a cluster with a representative sequence bearing a relatively high number of mutations compared to the parental sequence is considered relatively “younger” and more distant from the latter. Results are illustrated for the sequences of the P7 and P3 libraries in panels A and B, respectively, of Figure 6. Detailed analysis revealed that, for both libraries, common mutations lead to the fittest i.e. “largest” clusters. For example, mutations C138U and C148A –which favour the closing of the P4 stem –as well as mutations G307U and G31A –which are located in the P11 stem embedded within the hammerhead ribozyme for (+) and (−) polarities –are 4 substitutions that are clearly important for fitness. Two other such mutations, detected only in the P7 library, are the C118U substitution and the deletion of C117 –a cytosine located in the upper part of the P3 stem which bulges out from the structure, according to previous mapping data collected for another variant (PLMVd.034) using various ribonucleases [28]. We failed to detect either of these mutations in the P3 library since the cloning primers used annealed precisely to this region. Yet another key mutation important for fitness, detected in the P7 library, seems to be the deletion of U290. Surprisingly, this deletion is located right at the cleavage/ligation site. Although not found within representative sequences of the P3 library, this same mutation is born by 50.7% of P7 sequences. For unknown reasons, DHCS failed to recognize this mutation in the P3 library.

**Figure 7:**
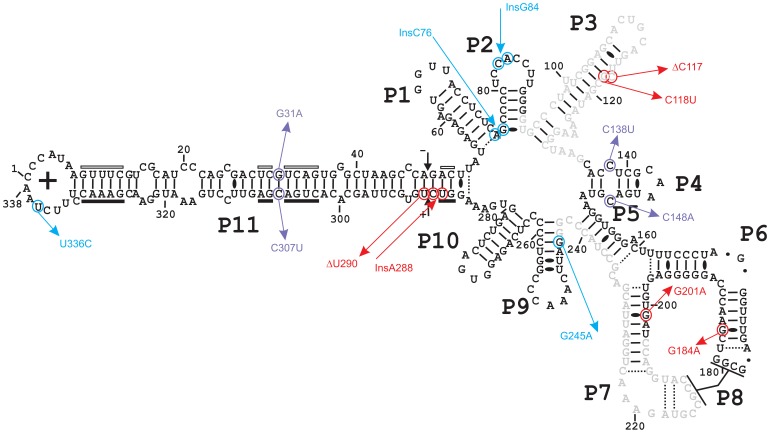
Key mutations detected in the representative sequence of each cluster. The key mutations that characterize the different clusters are depicted on the accepted secondary structure of the PLMVd (+) polarity strand. Mutations specifically identified in P7 library are in red, while those associated with P3 library are in blue. The purple mutations were found in both libraries. The black arrows indicate the hammerhead self-cleavage sites for the strands of both polarities. The black and open boxes below and above the P11 stem identify the regions that compose the core hammerhead nucleotides of the (+) and (−) polarity strands, respectively.

Lastly, different mutations arise from the fittest clusters leading to smaller ones of relatively inferior fitness. However, one cannot exclude the possibility that the relative fitness of emerging clusters was still evolving when our study RNA sample was collected 6 months post-infection. All of the key mutations presented in Figure 6 have been identified within the generally-accepted secondary structure of PLMVd (+), see Figure 7. The significance, as well as the effect, of these changes on replication or transport of PLMVd during infection requires further investigation.

In order to compare both libraries, the number of occurrences as a function of the position of the mutations was analyzed (see Figure S6). The same mutations were found to be present with virtually identical frequencies in both libraries.

### Mutations in the Vicinity of Hammerhead Self-cleaving Motifs

Analyses of the various mutations found within sequences displaying the most occurrences in each of the libraries (14,848 occurrences for P7 and 20,483 occurrences for P3), lead us to wonder whether such mutations conferred any replicative advantage over the parental sequence. The ribozyme hammerhead plays a central role in the viroid life cycle. Further analyses of mutations located in periphery of the ribozyme identified two modifications (deletion of U290 and substitution C307U) within the minimal hammerhead ribozyme of the (+) polarity strand as well as substitution G31A at the heart itself of the hammerhead ribozyme of the opposite strand.

**Figure 8:**
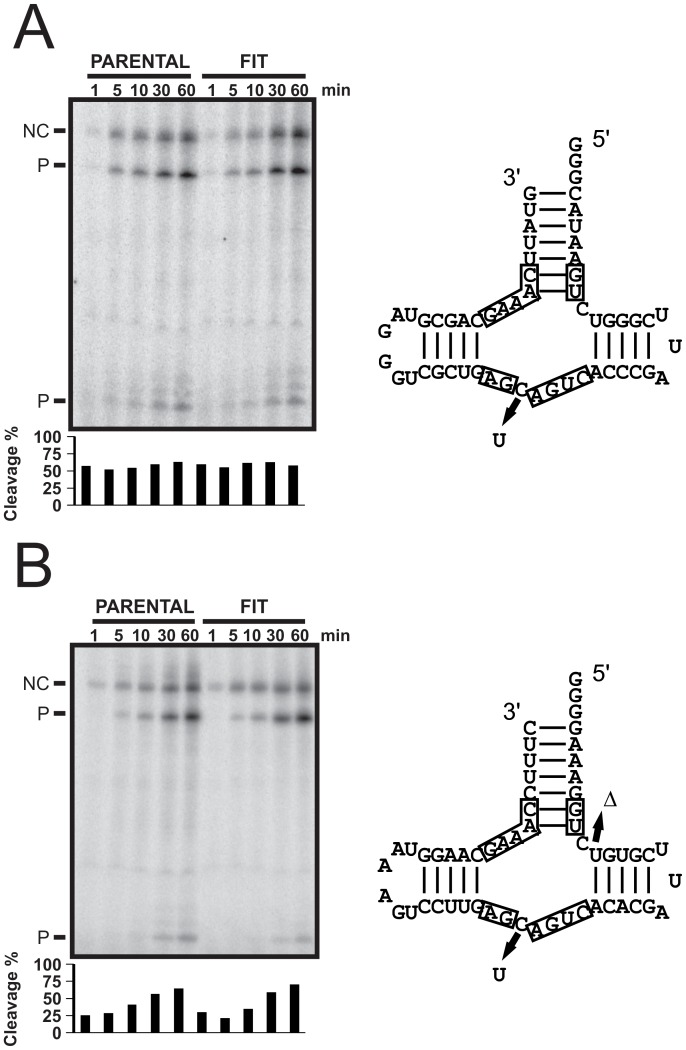
Cleavage efficiencies of the evolved self-cleaving hammerhead motifs. (A) A typical denaturing gel showing the cleavage of the (−) strand hammerhead ribozymes of the parental and most abundant sequence (FIT). In each case, aliquots were taken at 1, 5, 10, 30 and 60 minutes during the transcription of their respective DNA templates in the presence of [^32^P]UTP at 25^o^C. On the right side, the mutations relative to the parental sequence are depicted on the ribozyme structure. (B) Same as in (A), but for the (+) strand ribozymes.

We speculated whether the latter mutations not only influence self-cleavage activity, but also the actual copy number of a given sequence within the infected plant. Thus, in order to investigate whether or not mutations acquired by the parental sequence modulate variant fitness through self-cleavage, we designed a cleavage experiment comprising two chosen hammerhead sequences: the parental sequence (PLMVd.282) and the sequence of the variant with the most occurrences (the same sequence for both libraries). The hammerhead self-cleavage activities for both the (+) and (−) polarity strands of these variants were tested via *in vitro* transcription assays (Figure 8). Self-cleavage reaction during rather than post transcription is the most relevant window of investigation as it better reflects the cellular process of viroid replication. Experiments were performed in triplicate, and Figure 8 shows an example of denaturing gels for the hammerhead sequences of both polarities. Interestingly, when the hammerheads of both the parental sequence and the fittest-cluster representative sequence were compared, no significant differences were uncovered. All four sequences self-cleaved at ∼60–65% after 1 hour of incubation. Clearly, the level of self-cleavage alone cannot explain the level of occurrence of a given variant in the libraries. If this were the case, the strongest self-cleavage rate would have been observed for the fittest variant, and the weakest for the parental sequence.

## Discussion

The aim of this in-depth study was to explore the evolution of genetic variability of the circular conformers of the PLMVd quasispecies, six months post inoculation of a single sequence variant into a peach tree. Sequencing efforts focused exclusively on circular conformers, in order to obtain information from active variants only. Using two primer pairs, we gathered data from the entire viroid genome. To our knowledge, this is the first report of this scale using two distinct pairs of primers to assess the genetic heterogeneity of a viroid population.

Besides using KEC software for error correction, the novel approach described here uses the viroid’s highly-conserved hammerhead core nucleotides to determine a cut-off threshold for technical errors i.e. the systematic elimination of sequences harbouring artefact mutations introduced by RT and PCR reactions or HTS. This is the rationale underlying our use of hammerhead self-cleavage efficiency to remove reads containing technical errors. Since error-correction algorithms are not perfect (mutated hammerhead ribozymes not able to cleave still persisted after KEC correction), we added an additional layer of control by using a phenotypic character. Our hypothesis was that a read containing a mutated hammerhead motif that prevented self-cleavage, could not be biologically active in the cell. Such a read most likely resulted from an experimental artefact. Since a hammerhead sequence that was unable to self-cleave was found in a read with up to 4 occurrences, this level was arbitrarily used as the cut-off threshold. Thus, we eliminated all reads with 4 occurrences or less because of the hammerhead’s inability to cleave as it should despite being isolated from circular conformers. In doing so, not only did we remove from our analysis sequences bearing mutations in the hammerhead core, but also those sequences bearing mutations at any other position. To our knowledge, this is the first report of a design integrating a phenotypic criterion into the refinement of a deep-sequencing library.

Because the arbitrary threshold used does not take into account other aspects of the viroid replication cycle, it may be overly stringent. It is not impossible that a viroid possessing a relatively inefficient self-cleaving motif, might be particularly efficient at another step of the rolling-circle replication, such as the polymerisation or ligation step, or even that a viroid might possess RNA motifs that by binding to specific plant proteins enhance its transport throughout the infected plant. Because RT-PCR reactions and HTS introduce technical-error mutations, we carefully weighed the pros and cons of an arbitrarily set threshold. One important consideration was that the highly-conserved hammerhead motif sequence offered a biologically-sound, albeit stringent, means of determining such a threshold.

Despite this admittedly stringent threshold, nearly four thousand PLMVd sequences were retrieved. This is a relatively important yield, over an order of magnitude larger than previously published viroid-sequencing reports. Analysis of the libraries did not reveal the parental sequence originally used to inoculate the plant, even amongst the reads below the set threshold. This clearly demonstrates that the viroid sequence evolved over the 6-month period, despite being latent. A similar observation was recently made when a dimeric transcript of a PLMVd variant that induces peach calico was inoculated into GF-305 seedlings. Although small-scale sequencing was used, and only a small portion of the viroid genome was analyzed, the parental sequence was not recovered among the progeny [2]. The sequences retrieved here, using oligonucleotides complementary to the P7 region, unambiguously demonstrate that the P3 region contained a number of mutations. The P3 region cannot therefore be considered as highly-conserved for purposes of subsequent RT-PCR amplification and cloning. The P7 region, however, appeared to be more suited to this purpose, although certain mutations were found in this region in sequences of the P3 library. An ideal approach permits the linearization of the circular genome and the addition of linkers at both extremities prior to RT-PCR amplification, using oligonucleotides complementary to the external linker sequences. This type of strategy would permit the elucidation of the entire sequence of a viroid variant as no region of the viroid genome would be used to bind the primers.

Analysis of all PLMVd variants has led to several original observations. The large set of sequences (3,939 distinct variants) led us to assume that all 337 positions had the potential to be mutated. Paradoxically, however, we observed that ∼50% of positions remained perfectly conserved, including several small stretches as well as small motif reminiscent of a GNRA tetraloop (loop P10), which are the result of various selective pressures. Our observation is expected to stimulate experimental efforts, by us and others, to elucidate the biological mechanisms/functions associated with these conserved nucleotide sequences. Additionally, we found PLMVd variants that harboured approximately 50 mutations (Figure S2) compared to the parental sequence. This translates to ∼17% of sequence difference, clearly highlighting the potential of PLMVd as a quasispecies.

The pipeline reported here includes a divisive hierarchical clustering (DHCS) algorithm enabling model-based and alignment-free clustering of sequences. Although this algorithm has been used previously with protein sequences as input [22], this is the first report of its use for nucleic-acid clustering. We retrieved 7 clusters in both the P3 and P7 libraries, and the representative sequence of each cluster was identified. Most of the sequences within a given cluster are only 2 to 4 mutations away from its representative sequence (Figure 5). This clearly illustrates the effectiveness of the DHCS algorithm for the purpose of sequence clustering. This effectiveness is also highlighted by the identification of key mutations sufficiently powerful to discriminate between clusters through multiple alignments of the representative sequences of each cluster. The sequence clustering step also permitted the identification of mutations that lead to variations in cluster fitness, as measured by the total number of occurrences of sequences within a cluster. For example, the fittest cluster of the P7 library contained over 52,000 occurrences (cluster P7-OIO), while the fittest of the P3 library contained about 82,000 (cluster P3-OOI). Also, the most abundant sequence of each library is found within their respective fittest cluster. Interestingly, when sequences defining the viroid’s P3 and the P7 stem regions are removed, both sequences are identical. The most abundant sequences account for 10.8% and 13.2% of the data in the P7 and P3 libraries respectively.

Here, we show that the DHCS algorithm is a powerful tool for nucleic-acid sequence clustering of deep-sequencing data. Using this algorithm, we also identified representative sequences as well as their relative abundance and this, in turn, led us to identify key mutations in an original attempt to reconstruct the fascinating succession of events in the evolution of the viroid sequence.

Interestingly, the same mutations appear to be keys in both libraries. One interesting question is which of the fittest variants would have been be maintained? Because this study provides only a limited snapshot of the viroid progeny, 6 months post infection, we cannot answer this question. However, close examination of hammerhead self-cleaving motif efficiency provided certain clues. Self-cleavage catalytic efficiency during *in vitro* transcription did not correlate with the level of accumulated PLMVd variants (Figure 8). According to two studies conducted in the model plant *Arabidopsis thaliana*, viroid replication is necessary, but not sufficient, for infectivity [29], [30]. This suggests that the limiting step probably lies with the movement of the viroid within the plant. Clearly, the biological scenario is too intricately woven to turn only hammerhead self-cleavage, although selective pressures seem to have led to the conservation of this important RNA catalytic step.

In summary, we report the development of a reliable pipeline combining molecular biology and bioinformatics methods for the analysis of deep-sequencing data in order to study the genetic heterogeneity of a viroid population. Applying this procedure to PLMVd infected RNA samples using various viroid strains, or following various intervals of time post-infection, should help shed further light on the genetic evolution of a viroid population within a plant. To our knowledge, this is the most comprehensive report of its kind in terms of viroid sequence numbers. This study clearly demonstrates that viroid genomes are prone to high mutation rates within a single infected plant. In the case at hand, the most distant variants found in the libraries possessed a 17% variation level when compared to the parental sequence. This unexpected result brings into question the general assumption that 90% of an RNA should be conserved in order for it to be a quasispecies. Importantly, this study highlights the degree of sequence heterogeneity of PLMVd and its added value as a model viroid. Previous studies provided several interesting observations, however these were always based on a relatively limited number of sequences (i.e. <40 variants). Here, results provided from nearly 4,000 sequences have confirmed and provided an even more solid foundation to several of these conclusions. Further investigation is required in order to fully comprehend the mechanisms of viroid sequence variation. Using the pipeline proposed here, should help elucidate the population dynamics of these RNA quasispecies.

## Materials and Methods

### Plant Infection

The variant PLMVd.282 was dimerized and cloned as previously described [31]. The RNA was transcribed from a *SpeI*-digested plasmid using the TranscriptAidTM T7 High Yield Transcription Kit (Fermentas) and following the manufacturer’s instructions in order to generate the (+) polarity strand. The RNA was then purified by phenol:chloroform extraction followed by ethanol precipitation. The resulting RNA pellets were washed in 70% ethanol and then dissolved in loading buffer (95% formamide, 10 mM EDTA, pH 8.0, 0.025% xylene cyanol and 0.025% bromophenol blue). The samples were then fractionated through 5% denaturing polyacrylamide gels (PAGE, 19∶1 ratio of acrylamide to bisacrylamide) in buffer containing 45 mM Tris-borate, pH 7.5, 8 M urea and 2 mM EDTA. The dimeric RNA strand was extracted from the gel during a 16 h elution in an elution buffer containing 500 mM ammonium acetate, 10 mM EDTA and 0.1% SDS. After elution, the RNA was ethanol precipitated, washed with 70% ethanol, dried and dissolved in ultrapure water.

A healthy GF-305 peach tree obtained from a peach seedling was grown in greenhouse conditions prior to its slash inoculation with the dimeric RNA of PLMVd.282 dissolved in 50 mM KH_2_PO_4_.

### Purification of Circular Conformers from Total RNA

Total RNA was extracted from the leaves of the infected tree using Qiazol (Qiagen) according to the manufacturer’s protocol. The total RNA (10 µg) then DNase treated at 37^o^C for 90 min in a reaction containing 40 mM Tris pH 8.0, 10 mM MgSO_4_, 1 mM CaCl_2_ and 3 U of RQ1 DNAse (Promega) in a final volume of 20 µl. The DNA free RNA then was purified by phenol-chloroform extraction followed by ethanol precipitation. The resulting RNA pellet was washed in 70% ethanol, dried and finally dissolved in a 1∶1 mix of water and loading buffer (95% formamide, 10 mM EDTA, pH 8.0, 0.025% xylene cyanol and 0.025% bromophenol blue) prior to being fractionated through a 5% denaturing polyacrylamide gel (PAGE, 19∶1 ratio of acrylamide to bisacrylamide) in buffer containing 45 mM Tris-borate, pH 7.5, 8 M urea, and 2 mM EDTA. Along with the total RNA, a radiolabelled *in vitro* self-ligated PLMVd.282 (+) transcript (338 nt long) was also run on the gel. The gel band corresponding to the migration distance of this transcript was excised from the total RNA lane of the gel and the RNA eluted for 16 h in elution buffer described above. Following elution, the RNA was ethanol precipitated, washed with 70% ethanol, dried and dissolved in ultrapure water.

### Cloning of PLMVd Circular Genomes for Deep-sequencing

The purified RNA was reverse transcribed in the presence of either 20 µM of the P3rev primer (5′-TGCAGTGCTCCGAATAGGGCAN-3′), or 20 µM of the P7rev primer (5′-GTTTCTACGGCGGTACCTGN-3′), using Superscript III reverse transcriptase (100 U, Invitrogen). The purified RNA, the primer and dNTPs (0.5 mM) were combined in water and incubated at 65^o^C for 5 min prior to being snap cooled on ice for 2 min. The reverse transcription (RT) reactions were performed in 50 mM Tris-HCl pH 8.3, 75 mM KCl, 3 mM MgCl_2_, 5 mM DTT, RNaseOut (10 U, Invitrogen) and 5% DMSO at 50^o^C for 60 min.

After RT, the samples were PCR amplified using two primer pairs. The first pair, the P7F-P7 primer (5′-ccatctcatccctgcgtgtctccgactcag**AGACGCACTC**TGGATTACGACGTCTACCCGN-3′) and the P7R-Long primer (5′-cctatcccctgtgtgccttggcagtctcagGTTTCTACGGCGGTACCTGN-3′), was used on the cDNA produced with the P7rev primer in the previous RT. The second pair, the P3F-P3 primer (5′-ccatctcatccctgcgtgtctccgactcag**AGCACTGTAG**GTTCCCGATAGAAAGGCTAAN-3′) and the P3R-Long primer (5′-cctatcccctgtgtgccttggcagtctcagTGCAGTGCTCCGAATAGGGCAN-3′), was used on the cDNA produced with the P3rev primer. The small capitals nucleotides in the primers are the Titanium A and Titanium B specific sequences for the forward and reverse primers, respectively. The presence of these sequences is required by the 454-deep-sequencing facility. The bold uppercase nucleotides contain the sequence of the MIDBarcode, as both PCR products were multiplexed for the deep-sequencing reactions, while the uppercase nucleotides are specific to PLMVd. The PCR reactions were performed on the RT products in the presence of both sense and antisense primers (1 µM each) using Phusion DNA polymerase (2 U, New England BioLabs) as recommended by the manufacturer. The reactions were performed in Phusion HF buffer that contained 2.5 mM MgCl_2_, 3% DMSO and 0.2 mM dNTPs in a final volume of 100 µl and involved the steps: 98^o^C: 30 sec; 30 cycles of (10 sec at 98^o^C, 20 sec at 55^o^C, 15 sec at 72^o^C) followed by a final 7 min elongation step at 72^o^C. The PCR reactions were then separated on 1% agarose gels, the gel bands corresponding to the products of ∼350 bp were excised and the DNA purified using Spin-X centrifuge tube filters (Corning Incorporated). The purified DNA products were ethanol precipitated, washed and their pellets dissolved in ultrapure water. The samples were deep-sequenced at Genome Québec (Montréal, Canada) on a 454 GS-FLX Titanium pyrosequencing platform.

### Filtering of the Raw Data

Using a series of Perl scripts, unfiltered sequence reads from both sets (P7 and P3) were trimmed of both the 5′ and 3′ primer sequences. Reads that did not contain the primers were discarded, as were those containing uncertainties (i.e. N). Finally reads with lower than 70% identity to PLMVd were not kept for the following steps.

KEC software was used to correct and remove incorrect reads according to [17]. Mutations found in the core nucleotides of hammerhead ribozymes were identified following pairwise alignment [32] of each sequence with the parental sequence. The same mutation, found in reads of 1, 2 and 4 occurrences in the P7 library, was a uridine insertion between G12 and A13 of the hammerhead. The minimal hammerhead was identified and tested during *in vitro* transcription of the cognate DNA template (see below). Following the determination of the phenotypic threshold, sequences with 4 occurrences and less were removed from both P3 and P7 libraries.

Estimates of nucleotide diversity [21] were calculated for each library (containing reads of 5 occurrences or more) relatively to the inoculated sequence. Nucleotide diversity is defined as follows:
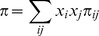
where 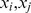 are the respective frequencies of the *i* ^th^, *j* ^th^ sequence and 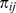 is the number of nucleotide differences between the *i* ^th^ and *j* ^th^ sequence. Since nucleotide diversity is computed relatively to the inoculated sequence, *i* = 1. 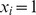 because the inoculated sequence is not retrieved. Nucleotide differences were computed by counting the number of different nucleotides from the pairwise alignment of P7 and P3 sequences [34] against the inoculated sequence. We found a nucleotide diversity of 1.39 for P3 and 1.60 for P7.

### Clustering

In order to cluster sequences, DHCS relies on both a new two-tier Markov Model and an iterative divisive hierarchical process. It initializes each division of the clustering process by assuming that sequences follow the first-order Markov model, and by using Fuzzy Multiple Correspondence Analysis. An advanced variable-order Conditional Probability Distribution model was then built for each of the two groups. The composition of each group was optimized using the Chi-square distance between each sequence and each group in order to move a sequence from one group to the other. The group division was iterated n times depending on the desired numbers of clusters and depths. The algorithm was also equipped with automatic stopping criteria. The DHCS algorithm effectively associates a sequence with the most similar group (for further details, see Xiong et al. [22]).

The DHCS was run on both the P7 and the P3 libraries. Three parameters were integrated: the Markov model’s order, the subsequence tree depth and the significance ratio which represented the minimal occurrence of a significant pattern (subsequence). The more the order and subsequence tree depth were increased, the greater the complexity. The order was set to 3, the tree depth to 4 and the significance ratio to 5. The software can be downloaded at: http://prospectus.usherbrooke.ca/dhcs/Download/Index.html.

### *In vitro* Self-cleavage Assays

DNA templates of the hammerhead sequences were prepared using a PCR-based strategy that included two oligodeoxynucleotides. The sense oligodeoxynucleotide contains the sequence of the T7 RNA polymerase promoter (5′- TAATACGACTCACTATAGGG -3′), while the antisense oligodeoxynucleotide contains both the inverse sequence of the hammerhead tested and the inverse sequence of the T7 RNA polymerase promoter at its 3′ end (see Figure S7 for the sequences of all of the oligodeoxynucleotides). For each self-cleaving sequence tested, 200 pmoles of both the sense and the antisense oligodeoxynucleotides were used in a filling reaction with *Pwo* DNA polymerase (Roche Diagnostics) containing 0.2 mM dNTPs, 20 mM Tris pH 8.8, 10 mM KCl, 10 mM (NH_4_)SO_4_, 0.1% Triton X-100 and 2 mM MgSO_4_ in a final volume of 100 µl. The reactions were subjected to 11 cycles of 30 sec at 94^o^C, 30 sec at 50^o^C, and 30 sec at 72^o^C. The resulting PCR products were ethanol precipitated, washed with 70% ethanol and then dissolved in ultrapure water prior to being used as templates for *in vitro* transcription. *In vitro* self-cleavage of the hammerhead ribozymes were monitored during transcription by performing the reactions in the presence of 10 µCi of [α-^32P^]GTP (3000 Ci/mmol; New England Nuclear), purified T7 RNA polymerase (5 µg), RNAseOut (10 U, Invitrogen), pyrophosphatase (0.005 U, Roche Diagnostics), and the PCR product (2 µM) in a buffer containing 80 mM HEPES-KOH pH 7.5, 24 mM MgCl_2_, 2 mM spermidine, 40 mM DTT and 5 mM of each NTP in a final volume of 50 µL at 25^o^C for 90 min. Upon completion, the reaction mixtures were treated with DNase RQ1 (Promega) at 37°C for 20 min, and the RNA then purified by phenol:chloroform extraction and ethanol precipitation. The resulting pellets were washed in 70% ethanol and dissolved in loading buffer (95% formamide, 10 mM EDTA, pH 8.0). The samples were then fractionated through 15% denaturing polyacrylamide gels (PAGE, 19∶1 ratio of acrylamide to bisacrylamide) in buffer containing 45 mM Tris-borate, pH 7.5, 8 M urea, and 2 mM EDTA. The reaction products were visualized by exposure to phosphor imaging screens and revealed on a Typhoon scanner (GE Healthcare).

In the case of the time course experiments, the transcription reactions were carried out at 25^o^C and 3 µl aliquots were withdrawn (at 1, 5, 10, 30 and 60 min) and immediately mixed with 15 µl of loading buffer in order to stop the reaction. Without further treatment, the samples were electrophoresed on a 15% denaturing polyacrylamide gel and exposed as described above.

## Supporting Information

Figure S1 **Analysis of the number of sequences of each occurrence found in both the P3 and P7 libraries.** This detailed analysis was performed on data following KEC correction.
(PDF)

Figure S2 **Number of mutations relative to the parental sequence.** For each library, all of the sequences over the threshold (>4 occurrences) were analyzed in terms of their numbers of mutations relative to the parental sequence.
(PDF)

Figure S3 **Cohesion and separation analysis of P3 and P7 datasets.**
(PDF)

Figure S4 **An alignment of the parental sequence with the representative sequences of each cluster of the P7 library.** Each key mutation is identified by a box.
(PDF)

Figure S5 **An alignment of the parental sequence with the representative sequences of each cluster of the P3 library.** Each key mutation is identified by a box.
(PDF)

Figure S6 **Types of mutations.** Graphs showing the number of occurrences of the different types of mutations, as well as their respective positions on the viroid’s genome for both libraries. The grey boxes cover the regions bound by the primers from which no genetic data is available.
(PDF)

Figure S7 **Oligonucleotides.** DNA oligonucleotides used as templates for the *in vitro* self-cleavage assays. The italic nucleotides (in 3′) represent the binding site for the T7 RNA polymerase promoter.
(PDF)
