# Correction: Deep-Sequencing of the Peach Latent Mosaic Viroid Reveals New Aspects of Population Heterogeneity

**DOI:** 10.1371/journal.pone.0096229

**Published:** 2014-04-21

**Authors:** The PLOS ONE Staff

Errors appear in Supplemental Figure 6 as well as the Acknowledgements section.

Supplemental Figure 6 is incorrect. Please see the corrected Supplemental Figure 6 here.

**Figure S6:**
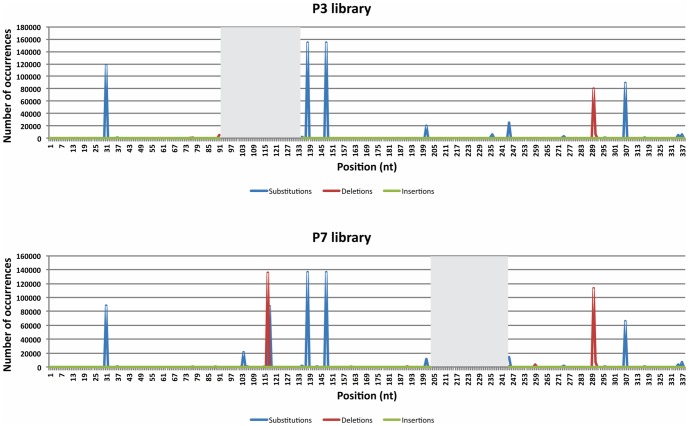
Types of mutations. Graphs showing the number of occurrences of the different types of mutations, as well as their respective positions on the viroid’s genome for both libraries. The grey boxes cover the regions bound by the primers from which no genetic data is available.

Please see the corrected author Acknowledgements section here.

The authors would like to acknowledge the contributions of Audrey Dubé and Olivier Parisi for technical assistance, in particular with the inoculations, Dominique Lévesque for the generation of plasmid pPD2-4, and Tengke Xiong for his assistance with the DHCS algorithm and programs. The sequence mining research of SW was partly funded by the Discovery Accelerator Supplements grant of Natural Sciences and Engineering Research Council of Canada (No. 396097-2010). R.J.N. is part of Centre de Recherche Clinique Étienne-Le Bel, a member of the Institute of Pharmacology of Sherbrooke, PROTEO (the Québec network for research on protein function, structure and engineering), GRASP (Groupe de Recherche Axé sur la Structure des Protéines) and is the recipient of a Junior I Researcher fellowship from the Fonds de Recherche du Québec en Santé (FRQ-S).
