# Supplementary material for: Deep-Sequencing of the Peach Latent Mosaic Viroid Reveals New Aspects of Population Heterogeneity
Source: PLoS One. 2014 Jan 30;9(1):e87297. doi: 10.1371/journal.pone.0087297 (PMC3907566; doi:10.1371/journal.pone.0087297)
Supplement: Figure S1 — Analysis of the number of sequences of each occurrence found in both the P3 and P7 libraries. This detailed analysis was performed on data following KEC correction. (PDF) [file pone.0087297.s001.pdf]

P7 Library		
**Number of occurrences**	**Number of different reads**	**Total number of reads**
**1**	11816	11816
**2**	2105	4210
**3**	983	2949
**4**	575	2300
**5**	322	1610
**6**	283	1698
**7**	166	1162
**8**	154	1232
**9**	108	972
**10**	110	1100
**11**	87	957
**12**	69	828
**13**	49	637
**14**	49	686
**15**	42	630
**16**	51	816
**17**	28	476
**18**	35	630
**19**	20	380
**20**	20	400
**21**	21	441
**22**	22	484
**23**	23	529
**24**	17	408
**25**	19	475
**26**	16	416
**27**	17	459
**28**	10	280
**29**	6	174
**30**	14	420
**31**	11	341
**32**	10	320
**33**	8	264
**34**	12	408
**35**	11	385
**36**	14	504
**37**	8	296
**38**	4	152
**39**	6	234
**40**	6	240

**41**	3	123
**42**	10	420
**43**	7	301
**44**	4	176
**45**	8	360
**46**	4	184
**47**	2	94
**48**	6	288
**49**	2	98
**50**	7	350
**51**	4	204
**52**	7	364
**53**	4	212
**54**	1	54
**55**	6	330
**56**	1	56
**57**	1	57
**58**	2	116
**59**	2	118
**60**	4	240
**61**	5	305
**62**	2	124
**63**	8	504
**64**	3	192
**65**	2	130
**66**	3	198
**67**	3	201
**68**	3	204
**69**	1	69
**70**	1	70
**71**	3	213
**72**	1	72
**73**	1	73
**74**	2	148
**75**	1	75
**76**	2	152
**77**	2	154
**78**	3	234
**79**	1	79
**80**	1	80
**81**	1	81
**82**	2	164
**83**	1	83

**84**	2	168
**85**	1	85
**87**	1	87
**90**	1	90
**91**	3	273
**92**	1	92
**94**	1	94
**96**	1	96
**97**	1	97
**98**	1	98
**99**	3	297
**101**	3	303
**102**	1	102
**104**	2	208
**105**	1	105
**107**	2	214
**108**	1	108
**110**	1	110
**112**	1	112
**113**	2	226
**114**	1	114
**115**	1	115
**118**	2	236
**119**	1	119
**121**	2	242
**122**	1	122
**123**	1	123
**125**	1	125
**127**	1	127
**133**	3	399
**134**	2	268
**136**	1	136
**137**	1	137
**138**	1	138
**139**	3	417
**140**	2	280
**141**	1	141
**143**	1	143
**147**	1	147
**148**	1	148
**151**	1	151
**152**	1	152
**154**	2	308

**156**	1	156
**158**	1	158
**159**	1	159
**161**	1	161
**162**	1	162
**165**	1	165
**166**	1	166
**167**	1	167
**170**	1	170
**173**	1	173
**174**	1	174
**178**	1	178
**180**	2	360
**184**	1	184
**185**	2	370
**190**	2	380
**195**	1	195
**197**	1	197
**202**	1	202
**209**	1	209
**214**	1	214
**217**	2	434
**219**	1	219
**234**	1	234
**238**	1	238
**242**	1	242
**247**	2	494
**249**	1	249
**252**	1	252
**259**	1	259
**263**	1	263
**278**	1	278
**287**	1	287
**299**	1	299
**304**	1	304
**314**	1	314
**322**	1	322
**341**	1	341
**343**	1	343
**345**	1	345
**363**	1	363
**367**	1	367
**384**	1	384

**386**	1	386
**392**	1	392
**404**	1	404
**408**	1	408
**410**	2	820
**424**	1	424
**430**	1	430
**435**	1	435
**439**	1	439
**443**	1	443
**452**	1	452
**462**	1	462
**473**	1	473
**520**	1	520
**529**	1	529
**555**	1	555
**557**	1	557
**569**	1	569
**620**	1	620
**648**	1	648
**649**	1	649
**687**	1	687
**715**	1	715
**883**	1	883
**897**	1	897
**927**	1	927
**939**	1	939
**1019**	1	1019
**1138**	1	1138
**1146**	1	1146
**1420**	1	1420
**1751**	1	1751
**1782**	1	1782
**1784**	1	1784
**1939**	1	1939
**2062**	1	2062
**2076**	1	2076
**2377**	1	2377
**2624**	1	2624
**2874**	1	2874
**2899**	1	2899
**4180**	1	4180
**4570**	1	4570

**5354**	1	5354
**6374**	1	6374
**11877**	1	11877
**14848**	1	14848
**TOTAL:**	**17622**	**158610**

P3 Library		
**Number of occurrences**	**Number of different reads**	**Total number of reads**
**1**	9350	9350
**2**	1763	3526
**3**	845	2535
**4**	495	1980
**5**	313	1565
**6**	219	1314
**7**	161	1127
**8**	122	976
**9**	97	873
**10**	99	990
**11**	63	693
**12**	69	828
**13**	44	572
**14**	50	700
**15**	32	480
**16**	27	432
**17**	34	578
**18**	19	342
**19**	15	285
**20**	24	480
**21**	21	441
**22**	20	440
**23**	12	276
**24**	22	528
**25**	7	175
**26**	12	312
**27**	12	324
**28**	9	252
**29**	14	406
**30**	10	300
**31**	8	248
**32**	11	352
**33**	7	231
**34**	8	272
**35**	7	245
**36**	7	252
**37**	6	222
**38**	9	342
**39**	9	351
**40**	9	360

**41**	6	246
**42**	7	294
**43**	5	215
**44**	5	220
**45**	4	180
**46**	7	322
**47**	5	235
**48**	6	288
**49**	3	147
**50**	5	250
**51**	4	204
**52**	6	312
**53**	1	53
**54**	3	162
**55**	2	110
**56**	2	112
**57**	5	285
**58**	4	232
**59**	3	177
**60**	4	240
**61**	2	122
**62**	8	496
**63**	2	126
**64**	4	256
**65**	2	130
**66**	2	132
**67**	6	402
**68**	3	204
**69**	4	276
**70**	5	350
**71**	2	142
**72**	1	72
**74**	5	370
**75**	2	150
**76**	2	152
**77**	4	308
**78**	5	390
**79**	2	158
**81**	2	162
**82**	2	164
**83**	1	83
**85**	2	170
**86**	2	172

**88**	2	176
**89**	1	89
**90**	1	90
**91**	2	182
**92**	5	460
**93**	1	93
**95**	2	190
**96**	1	96
**97**	2	194
**98**	3	294
**100**	1	100
**101**	1	101
**102**	1	102
**103**	4	412
**104**	3	312
**105**	2	210
**106**	2	212
**108**	2	216
**110**	2	220
**114**	1	114
**116**	1	116
**117**	2	234
**118**	2	236
**119**	1	119
**120**	1	120
**124**	1	124
**126**	1	126
**127**	2	254
**129**	2	258
**132**	1	132
**135**	2	270
**137**	1	137
**138**	1	138
**140**	1	140
**143**	1	143
**146**	1	146
**151**	1	151
**158**	1	158
**162**	3	486
**167**	1	167
**168**	1	168
**172**	1	172
**176**	1	176

**192**	1	192
**193**	1	193
**194**	1	194
**196**	1	196
**204**	1	204
**205**	1	205
**214**	1	214
**217**	1	217
**220**	1	220
**223**	1	223
**241**	1	241
**244**	1	244
**246**	1	246
**249**	1	249
**254**	1	254
**259**	1	259
**263**	1	263
**273**	1	273
**278**	1	278
**281**	1	281
**292**	1	292
**328**	1	328
**347**	1	347
**349**	1	349
**355**	1	355
**362**	1	362
**374**	1	374
**407**	1	407
**427**	1	427
**445**	1	445
**446**	1	446
**465**	1	465
**467**	1	467
**468**	1	468
**490**	1	490
**519**	1	519
**524**	1	524
**652**	1	652
**681**	1	681
**719**	1	719
**725**	1	725
**767**	1	767
**772**	1	772

**794**	2	1588
**826**	1	826
**827**	1	827
**872**	1	872
**896**	1	896
**945**	1	945
**998**	1	998
**1008**	1	1008
**1205**	1	1205
**1381**	1	1381
**1473**	1	1473
**1711**	1	1711
**1790**	1	1790
**1861**	1	1861
**1959**	1	1959
**2026**	1	2026
**2162**	1	2162
**2178**	1	2178
**2402**	1	2402
**2642**	1	2642
**2945**	1	2945
**7283**	1	7283
**7327**	1	7327
**8431**	1	8431
**8628**	1	8628
**17695**	1	17695
**20483**	1	20483
**TOTAL:**	**14358**	**172829**
