# Supplementary material for: Deep-Sequencing of the Peach Latent Mosaic Viroid Reveals New Aspects of Population Heterogeneity
Source: PLoS One. 2014 Jan 30;9(1):e87297. doi: 10.1371/journal.pone.0087297 (PMC3907566; doi:10.1371/journal.pone.0087297)
Supplement: Figure S2 — Number of mutations relative to the parental sequence. For each library, all of the sequences over the threshold (>4 occurrences) were analyzed in terms of their numbers of mutations relative to the parental sequence. (PDF) [file pone.0087297.s002.pdf]

Number of mutations relative to the parental sequence	Total number of reads (P7 Library)	Total number of reads (P3 Library)
1	0	0
2	0	7 327
3	2 901	22 506
4	15 574	43 731
5	21 529	47 292
6	29 761	24 473
7	35 267	7 888
8	21 942	1 267
9	7 813	62
10	1 290	55
11	97	74
12	23	45
13	14	54
14	50	32
15	29	58
16	37	22
17	116	19
18	118	17
19	125	10
20	129	0
21	73	0
22	18	0
23-35	0	0
36	0	9
37	0	10
38	0	8
39-45	0	0
46	0	10
47-49	0	0
50	0	52
51	0	32
**Total**	**136 906**	**155 053**
