# Supplementary material for: Deep-Sequencing of the Peach Latent Mosaic Viroid Reveals New Aspects of Population Heterogeneity
Source: PLoS One. 2014 Jan 30;9(1):e87297. doi: 10.1371/journal.pone.0087297 (PMC3907566; doi:10.1371/journal.pone.0087297)
Supplement: Figure S3 — Cohesion and separation analysis of P3 and P7 datasets. (PDF) [file pone.0087297.s003.pdf]

Cohesion and separation of P3 dataset

P3							
Model/Clusters	OOO	OOI	II	OIO	OII	IOO	IOI
ΔOOO	**1.98E+46**	6.72E+43	3.09E+43	4.26E+44	3.38E+36	2.28E+45	1.72E+45
ΔOOI	5.33E+46	**3.35E+48**	5.75E+45	2.14E+45	1.57E+41	1.04E+47	1.90E+47
ΔII	1.14E+43	5.70E+44	**1.70E+45**	1.04E+42	2.37E+43	2.39E+43	6.38E+44
ΔOIO	3.66E+42	1.49E+44	2.34E+43	**5.36E+45**	3.23E+43	1.80E+45	2.42E+45
ΔOII	2.07E+39	1.34E+41	5.62E+42	2.60E+46	**8.65E+47**	5.03E+45	1.63E+45
ΔIOO	1.15E+43	4.64E+44	2.18E+44	2.10E+42	4.81E+43	**1.97E+46**	2.48E+45
ΔIOI	4.26E+42	2.68E+44	1.23E+44	3.02E+41	7.94E+42	2.62E+44	**1.38E+46**

Cohesion and separation of P7 dataset

P7							
Model/Clusters	OO	OIO	OII	IIO	III	IOO	IOI
ΔOO	**1.70E+50**	1.39E+49	8.98E+47	7.24E+47	2.85E+49	5.80E+47	1.84E+46
ΔOIO	1.20E+51	**7.40E+52**	3.37E+51	3.76E+51	1.17E+51	3.83E+45	4.03E+44
ΔOII	7.54E+49	1.03E+50	**3.06E+51**	5.71E+49	5.29E+49	1.21E+43	2.73E+44
ΔIIO	5.19E+46	2.41E+48	2.02E+47	**1.16E+51**	9.09E+48	5.16E+43	7.44E+41
ΔIII	2.79E+46	1.88E+48	9.15E+46	1.31E+47	**2.05E+49**	3.90E+44	1.01E+43
ΔIOO	5.20E+49	1.46E+45	1.01E+44	9.23E+43	3.84E+49	**2.09E+51**	2.14E+50
ΔIOI	1.51E+48	1.12E+42	5.15E+43	1.63E+42	9.05E+47	1.11E+49	**1.19E+50**
