# Supplementary material for: Deep-Sequencing of the Peach Latent Mosaic Viroid Reveals New Aspects of Population Heterogeneity
Source: PLoS One. 2014 Jan 30;9(1):e87297. doi: 10.1371/journal.pone.0087297 (PMC3907566; doi:10.1371/journal.pone.0087297)
Supplement: Figure S4 — An alignment of the parental sequence with the representative sequences of each cluster of the P7 library. Each key mutation is identified by a box. (PDF) [file pone.0087297.s004.pdf]

```
                                                                  InsA288   ΔU290
Parent seq         GAUUCAAACCCGGUCCCCCUCAGAGGUGACUCUGAGUGAAAGGUCUGUGCUUAGCACACU
P7_III_189;66;2    GAUUCAAACCCGGUCCCCCUCAGAGGUGACUCUGAGUGAAAGGUACGUGCUUAGCACACU
P7_IIO_14;1784;    GAUUCAAACCCGGUCCCCCUCAGAGGUGACUCUGAGUGAAAGGU-CGUGCUUAGCACACU
P7_IOI_2;11877;    GAUUCAAACCCGGUCCCCCUCAGAGGUGACUCUGAGUGAAAGGU-CGUGCUUAGCACACU
P7_IOO_6;4180;2    GAUUCAAACCCGGUCCCCCUCAGAGGUGACUCUGAGUGAAAGGU-CGUGCUUAGCACACU
P7_OII_4;5354;2    GAUUCAAACCCGGUCCCCCUCAGAGGUGACUCUGAGUGAAAGGU-CGUGCUUAGCACACU
P7_OIO_1;14848;    GAUUCAAACCCGGUCCCCCUCAGAGGUGACUCUGAGUGAAAGGU-CGUGCUUAGCACACU
P7_OO_315;37;29    GAUUCAAACCCGGUCCCCCUCAGAGGUGACUCUGAGUGAAAGGU-CGUGCUUAGCACACU
                   ******************************************** .**************

                      C307U
Parent seq         GACGAGUUCCUGAAAUGGAACGAAACCUUUCUAACCCAUAAGUUUCGUCGCAUCCCAGCG
P7_III_189;66;2    GAUGAGUUCCUGAAAUGGAACGAAACCUUUCUAACCCAUAAGUUUCGUCGCAUCCCAGCG
P7_IIO_14;1784;    GAUGAGUUCCUGAAAUGGAACGAAACCUUUCUAACCCAUAAGUUUCGUCGCAUCCCAGCG
P7_IOI_2;11877;    GACGAGUUCCUGAAAUGGAACGAAACCUUUCUAACCCAUAAGUUUCGUCGCAUCCCAGCG
P7_IOO_6;4180;2    GACGAGUUCCUGAAAUGGAACGAAACCUUUCUAACCCAUAAGUUUCGUCGCAUCCCAGCG
P7_OII_4;5354;2    GAUGAGUUCCUGAAAUGGAACGAAACCUUUCUAACCCAUAAGUUUCGUCGCAUCCCAGCG
P7_OIO_1;14848;    GAUGAGUUCCUGAAAUGGAACGAAACCUUUCUAACCCAUAAGUUUCGUCGCAUCCCAGCG
P7_OO_315;37;29    GAUGAGUUCCUGAAAUGGAACGAAACCUUUCUAACCCAUAAGUUUCGUCGCAUCCCAGCG
                   **.*********************************************************

                        G31A
Parent seq         ACUCGUCAGUGGGCUAAGCCCAGACUUAUGAGAGAGUGGUUACCUCUCAGCCCCUCCACC
P7_III_189;66;2    ACUCAUCAGUGGGCUAAGCCCAGACUUAUGAGAGAGUGGUUACCUCUCAGCCCCUCCACC
P7_IIO_14;1784;    ACUCAUCAGUGGGCUAAGCCCAGACUUAUGAGAGAGUGGUUACCUCUCAGCCCCUCCACC
P7_IOI_2;11877;    ACUCGUCAGUGGGCUAAGCCCAGACUUAUGAGAGAGUGGUUACCUCUCAGCCCCUCCACC
P7_IOO_6;4180;2    ACUCGUCAGUGGGCUAAGCCCAGACUUAUGAGAGAGUGGUUACCUCUCAGCCCCUCCACC
P7_OII_4;5354;2    ACUCAUCAGUGGGCUAAGCCCAGACUUAUGAGAGAGUGGUUACCUCUCAGCCCCUCCACC
P7_OIO_1;14848;    ACUCAUCAGUGGGCUAAGCCCAGACUUAUGAGAGAGUGGUUACCUCUCAGCCCCUCCACC
P7_OO_315;37;29    ACUCAUCAGUGGGCUAAGCCCAGACUUAUGAGAGAGUGGUUACCUCUCAGCCCCUCCACC
                   ****.*******************************************************

                                               ΔC117  C118U               C138U
Parent seq         UUGGGGUGCCCUAUUCGGAGCACUGCAGUUCCCGAUAGAAAGGCUAAGCACCUCGCAAUG
P7_III_189;66;2    UUGGGGUGCCCUAUUCGGAGCACUGCAGUU-UCGAUAGAAAGGCUAAGCACUUCGCAAUG
P7_IIO_14;1784;    UUGGGGUGCCCUAUUCGGAGCACUGCAGUU-UCGAUAGAAAGGCUAAGCACUUCGCAAUG
P7_IOI_2;11877;    UUGGGGUGCCCUAUUCGGAGCACUGCAGUU-CCGAUAGAAAGGCUAAGCACUUCGCAAUG
P7_IOO_6;4180;2    UUGGGGUGCCCUAUUCGGAGCACUGCAGUU-UCGAUAGAAAGGCUAAGCACUUCGCAAUG
P7_OII_4;5354;2    UUGGGGUGCCCUAUUCGGAGCACUGCAGUU-CCGAUAGAAAGGCUAAGCACUUCGCAAUG
P7_OIO_1;14848;    UUGGGGUGCCCUAUUCGGAGCACUGCAGUU-UCGAUAGAAAGGCUAAGCACUUCGCAAUG
P7_OO_315;37;29    UUGGGGUGCCCUAUUCGGAGCACUGCAGUU-UCGAUAGAAAGGCUAAGCACUUCGCAAUG
                   ****************************** .*******************.********

                     C148A                          G184A            G201A
Parent seq         ACGUAAGGUGGGACUUUUCCCUAGGGUUUGAGCGGUCGAACCCAGGGGGAGUGUGAU
P7_III_189;66;2    AAGUAAGGUGGGACUUUUCCCUAGGGUUUGAGCGGUCGAACCCAGGGGGAGUGUGAU
P7_IIO_14;1784;    AAGUAAGGUGGGACUUUUCCCUAGGGUUUGAGCGGUCGAACCCAGGGGGAGUGUAAU
P7_IOI_2;11877;    AAGUAAGGUGGGACUUUUCCCUAGGGUUUGAGCGGUCGAACCCAGGGGGAGUGUGAU
P7_IOO_6;4180;2    AAGUAAGGUGGGACUUUUCCCUAGGGUUUGAGCGGUCGAACCCAGGGGGAGUGUGAU
P7_OII_4;5354;2    AAGUAAGGUGGGACUUUUCCCUAGGGUUUGAGCGGUCGAACCCAGGGGGAGUGUGAU
P7_OIO_1;14848;    AAGUAAGGUGGGACUUUUCCCUAGGGUUUGAGCGGUCGAACCCAGGGGGAGUGUGAU
P7_OO_315;37;29    AAGUAAGGUGGGACUUUUCCCUAGGGUUUGAGCGGUCAAACCCAGGGGGAGUGUGAU
                   *.***********************************.****************.**
```
