# Supplementary material for: Deep-Sequencing of the Peach Latent Mosaic Viroid Reveals New Aspects of Population Heterogeneity
Source: PLoS One. 2014 Jan 30;9(1):e87297. doi: 10.1371/journal.pone.0087297 (PMC3907566; doi:10.1371/journal.pone.0087297)
Supplement: Figure S5 — An alignment of the parental sequence with the representative sequences of each cluster of the P3 library. Each key mutation is identified by a box. (PDF) [file pone.0087297.s005.pdf]

```
                         C138U     C148A
Parent seq         CACCUCGCAAUGACGUAAGGUGGGACUUUUCCCUAGGGUUUGAGCGGUCGAACCCAGGGG
P3_II_349;34;29    CACUUCGCAAUGAAGUAAGGUGGGACUUUUCCCUAGGGUUUGAGCGGUCGAACCCAGGGG
P3_IOI_67;204;2    CACUUCGCAAUGAAGUAAGGUGGGACUUUUCCCUAGGGUUUGAGCGGUCGAACCCAGGGG
P3_IOO_83;140;2    CACUUCGCAAUGAAGUAAGGUGGGACUUUUCCCUAGGGUUUGAGCGGUCGAACCCAGGGG
P3_OII_5;7327;2    CACUUCGCAAUGAAGUAAGGUGGGACUUUUCCCUAGGGUUUGAGCGGUCGAACCCAGGGG
P3_OIO_39;467;2    CACUUCGCAAUGAAGUAAGGUGGGACUUUUCCCUAGGGUUUGAGCGGUCGAACCCAGGGG
P3_OOI_2;17695;    CACUUCGCAAUGAAGUAAGGUGGGACUUUUCCCUAGGGUUUGAGCGGUCGAACCCAGGGG
P3_OOO_7;2945;2    CACUUCGCAAUGAAGUAAGGUGGGACUUUUCCCUAGGGUUUGAGCGGUCGAACCCAGGGG
                   ***.********* **********************************************

                                                                      G245A
Parent seq         GAGUGUGAUCCAGGUACCGCCGUAGAAACUGGAUUACGACGCCUACCCGGGAUUCAAACC
P3_II_349;34;29    GAGUGUGAUCCAGGUACCGCCGUAGAAACUGGAUUACGACGCCUACCCGGGAUUCAAACC
P3_IOI_67;204;2    GAGUGUGAUCCAGGUACCGCCGUAGAAACUGGAUUACGACGCCUACCCGGGAUUCAAACC
P3_IOO_83;140;2    GAGUGUGAUCCAGGUACCGCCGUAGAAACUGGAUUACGACGCCUACCCGGGAUUCAAACC
P3_OII_5;7327;2    GAGUGUGAUCCAGGUACCGCCGUAGAAACUGGAUUACGACGCCUACCCGGGAUUCAAACC
P3_OIO_39;467;2    GAGUGUGAUCCAGGUACCGCCGUAGAAACUGGAUUACGACGCCUACCCGGAAUUCAAACC
P3_OOI_2;17695;    GAGUGUGAUCCAGGUACCGCCGUAGAAACUGGAUUACGACGCCUACCCGGGAUUCAAACC
P3_OOO_7;2945;2    GAGUGUGAUCCAGGUACCGCCGUAGAAACUGGAUUACGACGCCUACCCGGAAUUCAAACC
                   **************************************************.*********

                                                                         C307U
Parent seq         CGGUCCCCCUCAGAGGUGACUCUGAGUGAAAGGUCUGUGCUUAGCACACUGACGAGUUCC
P3_II_349;34;29    CGGUCCCCCUCAGAGGUGACUCUGAGUGAAAGGUCUGUGCUUAGCACACUGAUGAGUUCC
P3_IOI_67;204;2    CGGUCCCCCUCAGAGGUGACUCUGAGUGAAAGGUCUGUGCUUAGCACACUGAUGAGUUCC
P3_IOO_83;140;2    CGGUCCCCCUCAGAGGUGACUCUGAGUGAAAGGUCUGUGCUUAGCACACUGAUGAGUUCC
P3_OII_5;7327;2    CGGUCCCCCUCAGAGGUGACUCUGAGUGAAAGGUCUGUGCUUAGCACACUGACGAGUUCC
P3_OIO_39;467;2    CGGUCCCCCUCAGAGGUGACUCUGAGUGAAAGGUCUGUGCUUAGCACACUGACGAGUUCC
P3_OOI_2;17695;    CGGUCCCCCUCAGAGGUGACUCUGAGUGAAAGGUCUGUGCUUAGCACACUGAUGAGUUCC
P3_OOO_7;2945;2    CGGUCCCCCUCAGAGGUGACUCUGAGUGAAAGGUCUGUGCUUAGCACACUGAUGAGUUCC
                   ****************************************************.*******

                                          U336C                         G31A
Parent seq         UGAAAUGGAACGAAACCUUUCUAACCCAUAAGUUUCGUCGCAUCCCAGCGACUCGUCAGU
P3_II_349;34;29    UGAAAUGGAACGAAACCUUUCUAACCCAUAAGUUUCGUCGCAUCCCAGCGACUCAUCAGU
P3_IOI_67;204;2    UGAAAUGGAACGAAACCUUUCUAACCCAUAAGUUUCGUCGCAUCCCAGCGACUCAUCAGU
P3_IOO_83;140;2    UGAAAUGGAACGAAACCUUUCCAACCCAUAAGUUUCGUCGCAUCCCAGCGACUCAUCAGU
P3_OII_5;7327;2    UGAAAUGGAACGAAACCUUUCUAACCCAUAAGUUUCGUCGCAUCCCAGCGACUCGUCAGU
P3_OIO_39;467;2    UGAAAUGGAACGAAACCUUUCUAACCCAUAAGUUUCGUCGCAUCCCAGCGACUCGUCAGU
P3_OOI_2;17695;    UGAAAUGGAACGAAACCUUUCUAACCCAUAAGUUUCGUCGCAUCCCAGCGACUCAUCAGU
P3_OOO_7;2945;2    UGAAAUGGAACGAAACCUUUCUAACCCAUAAGUUUCGUCGCAUCCCAGCGACUCAUCAGU
                   *********************.********************************.*****

                                                          InsC76          InsG84
Parent seq         GGGCUAAGCCCAGACUUAUGAGAGAGUGGUUACCUCUCA-GCCCCUCC-ACCUUGGG
P3_II_349;34;29    GGGCUAAGCCCAGACUUAUGAGAGAGUGGUUACCUCUCA-GCCCCUCCGACCUUGGG
P3_IOI_67;204;2    GGGCUAAGCCCAGACUUAUGAGAGAGUGGUUACCUCUCACGCCCCUCC-ACCUUGGG
P3_IOO_83;140;2    GGGCUAAGCCCAGACUUAUGAGAGAGUGGUUACCUCUCA-GCCCCUCC-ACCUUGGG
P3_OII_5;7327;2    GGGCUAAGCCCAGACUUAUGAGAGAGUGGUUACCUCUCA-GCCCCUCC-ACCUUGGG
P3_OIO_39;467;2    GGGCUAAGCCCAGACUUAUGAGAGAGUGGUUACCUCUCA-GCCCCUCC-ACCUUGGG
P3_OOI_2;17695;    GGGCUAAGCCCAGACUUAUGAGAGAGUGGUUACCUCUCA-GCCCCUCC-ACCUUGGG
P3_OOO_7;2945;2    GGGCUAAGCCCAGACUUAUGAGAGAGUGGUUACCUCUCA-GCCCCUCC-ACCUUGGG
                   *************************************** ******** ********
```
