# Supplementary material for: Deep-Sequencing of the Peach Latent Mosaic Viroid Reveals New Aspects of Population Heterogeneity
Source: PLoS One. 2014 Jan 30;9(1):e87297. doi: 10.1371/journal.pone.0087297 (PMC3907566; doi:10.1371/journal.pone.0087297)
Supplement: Figure S6 — Types of mutations. Graphs showing the number of occurrences of the different types of mutations, as well as their respective positions on the viroid’s genome for both libraries. The grey boxes cover the regions bound by the primers from which no genetic data is available. (PDF) [file pone.0087297.s006.pdf]

>Mutated core hammerhead
GAAAGGTTTACGTTTCCATTTCTAGGAACTCATCAGTGTGCTAAGCACGACCTTTCCCC*TATAGTGAGTCGTATTA*

>Parental PLMVd.282 (+) polarity
GAAAGGTTTCGTTCCATTTCAGGAACTCGTCAGTGTGCTAAGCACAGACCTTTCCCC*TATAGTGAGTCGTATTA*

>Parental PLMVd.282 (-) polarity
CATAAGTTTCGTCGCATCCCAGCGACTCGTCAGTGGGCTAAGCCCAGACTTATGCCC*TATAGTGAGTCGTATTA*

>FIT (+) polarity
GAAAGGTTTCGTTCCATTTCAGGAACTCATCAGTGTGCTAAGCACGACCTTTCCCC*TATAGTGAGTCGTATTA*

>FIT (-) polarity
CATAAGTTTCGTCGCATCCCAGCGACTCATCAGTGGGCTAAGCCCAGACTTATGCCC*TATAGTGAGTCGTATTA*
